# Polydopamine/polyvinyl alcohol/graphene oxide transition layer for enhancing adhesive performance of HA coating on C_f_/C composites prepared by hydrothermal electrodeposition/hydrothermal treatment

**DOI:** 10.1007/s10856-025-06922-2

**Published:** 2025-08-20

**Authors:** Shaoqing Chen, Caiqin Liang, Pengyin Li, Chun Liu, Xierong Zeng, Xinbo Xiong, Xinye Ni

**Affiliations:** 1 The Affiliated Changzhou No. 2 People’s Hospital of Nanjing Medical University, Changzhou Medical Center, Nanjing Medical University, Changzhou, China; 2 Shenzhen Key Laboratory of Special Functional Materials, College of Materials, Shenzhen University, Shenzhen, China

## Abstract

Hydroxyapatite (HA) coatings on carbon fiber-reinforced carbon (C_f_/C) composites hold promise for orthopedic implants. However, the interface between HA and C_f_/C is prone to delamination, limiting its application. To address this, a polydopamine (PDA)-polyvinyl alcohol (PVA)-graphene oxide (GO) transition layer was introduced to reinforce and toughen HA coatings on C_f_/C composites (PDA-PVA-GO/C_f_/C) via hydrothermal electro-deposition/post-hydrothermal treatment. For comparison, the PDA and PDA/PVA transition layers were also prepared on C_f_/C, designated as PDA/C_f_/C and PDA-PVA/C_f_/C, respectively. The precursor and transformed coatings obtained were monetite and HA. XRD analyses revealed that PDA and PVA infiltrated the monetite lattice without affecting the HA lattice parameters. Remarkably, scratch tests demonstrated that the HA/PVD-PVA-GO coating on C_f_/C exhibited a dense configuration and compact interfacial structure, achieving a maximum critical load of 51.5 N, surpassing other reported electrochemically prepared HA coatings. Moreover, scratch tests indicated a more homogeneous scratch pattern with no sudden delamination of the coating from the matrix. In vitro assessments revealed that all HA coatings with the transition layer exhibited enhanced bioactivity and cell compatibility compared with HA alone. In particular, PDA/PVA/GO-C_f_/C exhibited the best superior efficacy in promoting the proliferation of mouse embryonic osteoblast precursor (MC3T3-E1) cells and significantly increased Alkaline phosphatase (ALP) production in rat bone marrow mesenchymal stem cells (BMSCs). These findings underscore the potential of PDA-PVA-GO/C_f_/C as a promising biomaterial for bone regeneration.

**Graphical Abstract:**

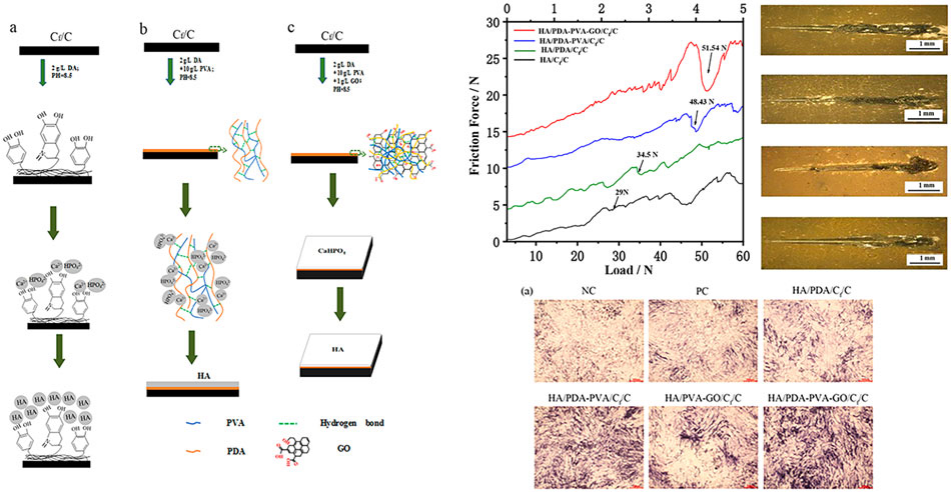

## Introduction

Carbon, a constituent of the human body, finds extensive use in orthopedic applications such as bones, tendons, blood vessels, tooth roots, and heart valves, owing to its excellent safety profile. Carbon fiber-reinforced carbon (C_f_/C) composites exhibit superior mechanical properties, exceptional corrosion resistance, and excellent biocompatibility [1, 2]. Notably, their modulus of elasticity closely resembles that of human bone, mitigating stress shielding [3]. However, their biological inertness and propensity to release carbon debris post-implantation hinder their integration with surrounding tissues [4]. In contrast, hydroxyapatite (HA) demonstrates remarkable bioactivity and osteoconductivity, facilitating rapid bone formation and tissue integration [5, 6]. Nevertheless, its inherent mechanical weaknesses and brittleness pose challenges for biological applications [7]. Hence, the deposition of HA coatings on C_f_/C composites presents an opportunity to merge outstanding mechanical properties with biological activity [3, 8].

Hydrothermal electrodeposition (HED) stands out as a favored technique for HA coating on bioinert conductive materials like metals or C_f_/C composites, offering precise control over coating composition and microstructure, alongside high bioactivity [9–11]. However, delamination at the HA-C_f_/C interface, primarily due to weak hydrogen bonding and physical/mechanical bonding, limits the utility of HA-coated C_f_/C composites in orthopedic applications. Thermal expansion coefficient disparities between the HA coating and the C_f_/C substrate, coupled with diverse carbon structures on the composites, complicate this challenge. Introducing a transition layer between HA and C_f_/C composites emerges as an effective strategy to address this issue. Various materials, including SiC [12–14], carbon foam [15], carbon nanotubes (CNTs) [16], graphene oxide (GO) [17–20], and TC4 [21], have been explored for this purpose. For instance, Li et al. utilized SiC as a transition layer to fabricate a Si-HA/SiC composite coating on C_f_/C. Although the commendable biological activity of the coating was noted, its bonding properties with the C_f_/C substrate were not commented on [12]. In another study, Zhang et al. employed a novel “tree-planting” interface, composed of micro-oxidized C–C substrate holes and CNTs, as a transition zone for HA and C_f_/C composites. This innovative approach yielded a remarkable bond strength of 11.14 ± 0.78 MPa for the HA coating on C_f_/C, making a substantial 181.3% increase compared to coatings on C–C without the interface (3.96 ± 0.30 MPa) [8]. GO emerges as a popular candidate for reinforcing and toughening HA coatings due to its abundance in oxygen-containing functional groups [21]. Guan et al. introduced a SiC inner layer and a GO middle layer, discovering that the resulting SiC-GO-HA coating achieved a bond strength of 12.56 MPa, signifying a 160.58% enhancement over single-layer HA coatings [17]. Similarly, Zhang et al. explored the application of an electrophoretically deposited GO transition layer on C_f_/C, followed by simultaneous electrodeposition of GO and Mg-substituted hydroxyapatite on GO-modified C_f_/C composites. This dual-layer coating exhibited a bond strength of 7.4 MPa, representing an 80.49% improvement over single-layered MH coatings lacking GO [18]. These investigations collectively underscore a recurring challenge: the current suite of transition materials fails to deliver HA coatings on C_f_/C with optimal adhesion performance, characterized by high bonding strength and resistance to delamination. Addressing this limitation remains paramount for advancing their utilization in orthopedic applications.

Biopolymers have emerged as promising candidates for transition layer materials, facilitating the bonding of HA coatings to C_f_/C composites. Previous research utilized a blend of polyvinyl alcohol (PVA)/GO as a transition layer to C_f_/C substrates, enabling the subsequent application of HA coatings. Scratch tests demonstrated a notable bond strength, reaching a critical load of 41.04 N [22]. Notably, even under the critical load, the C_f_/C substrate with an HA-PVA/GO coating exhibited no signs of delamination. However, the preparation process suffered from instability and difficulty in control, leading to the exposure of localized failure surfaces in the C_f_/C substrate. Additionally, the utility of PVA in orthopedic implants has been constrained by its comparatively low mechanical strength. To address these limitations, researchers have explored strategies to enhance the mechanical properties of PVA, including incorporating other biopolymers or organic minerals such as polylactide [23], polydopamine (PDA) [24], gelatin [25], chitosan [26], collagen [27], alginate [28], HA, or other calcium phosphates [29]. Among these, dopamine, inspired by mussel adhesive protein, has garnered significant interest. With the help of catechol and amine functional groups, dopamine could self-polymerize under alkaline aqueous conditions to form PDA, which can strongly adhere to a wide array of substrates, including polymers, metals, glasses, ceramics, and carbon materials, without necessitating pre-treatment [27]. The strong interfacial adhesion of PDA extends to GO nanosheets, imparting hydrophilicity and reactivity to residual catechol groups and the oxidized quinone form of catechol. Additionally, π-π interactions between PDA and the graphitic domains of GO enhance interfacial bonding, further reinforcing the GO nanosheets [30]. Importantly, PDA exhibits high mechanical properties in liquid media and demonstrates excellent compatibility with PVA, attributed to the ample free volume of PDA chains and robust hydrogen bonding [31]. Consequently, this compatibility aids in improving the mechanical performance of PVA and mitigates its dissolution in aqueous solutions. Moreover, PDA can complex with metal ions such as Ca^2+^ through its polyphenol functional groups. These complexes serve as nucleation sites for HA crystal formation, thus facilitating subsequent HA deposition [32].

Based on the aforementioned analyses, the present study assumes the implementation of a PDA-PVA-GO transition layer to fortify and enhance the interface between the HA coating and the C_f_/C matrix. This is achieved through a combination of HED and hydrothermal post-treatment, aimed at addressing the limitations in the mechanical performance of HA coatings on C_f_/C composites. Transition layers with and without PDA, and PDA-PVA on C_f_/C, were additionally prepared to facilitate comparison. The study extensively investigates the coatings’ physical and chemical properties, adhesion properties, in vitro compatibility, and osteogenetic effects.

## Experimental methods

### Preparation and treatment of C_f_/C

The C_f_/C bulk material, sourced from Shanghai University, was cut into several sheets measuring 10 × 10 × 2 mm^3^. These bare C_f_/C samples were polished with abrasive paper, followed by ultrasonic cleaning in a sequence of acetone (Sanpu Chemical Reagents Co., Ltd., China, AR), alcohol (Sinopharm Chemical Reagent Co., Ltd., China), and deionized water. Subsequently, the samples were subjected to hydrothermal modification in an autoclave using a 1 M (NH_4_)_2_ S_2_O_8_ (Guangzhou Chemical Reagent Factory, China, AR) solution at 433 K for 12 h. Finally, the C_f_/C sheets were ultrasonically rinsed with distilled water and dried in preparation for the subsequent deposition of calcium phosphate (CaP) coating.

### Preparation of PDA, PDA/PVA, and PDA/PVA/GO layers on C_f_/C

First, three solutions were prepared in the following steps: (1) Dopamine hydrochloride (DA) powder (obtained from Shanghai Macklin) was dissolved in deionized water to prepare a 2 g/L DA solution; (2) DA at a fixed concentration of 2 g/L was dissolved in a 10 g/L PVA (Shanghai Aladdin Biochemical Technology Co., Ltd., China, AR, powder) solution to produce a DA-PVA mixed solution; (3) a mixed solution with the concentrations of DA, PVA, and GO (Shuiheng Graphene Technology Co., Ltd., China, >99 wt%, severe oxidation, with oxygen content exceeding 50%) was 2 g/L, 10 g/L, and 1 g/L, respectively. PVA powder was mixed with deionized water at a mass ratio of 1:10, followed by dissolution in an oil bath at 90 °C to obtain a homogeneous PVA solution. GO was dispersed in deionized water and subjected to ultrasonication for 40 min to form a stable yellowish-brown suspension. The PVA solution was then combined with the GO suspension at a volume ratio of 5:1, followed by an additional 40-min ultrasonication to yield a uniform golden-yellow PVA/GO composite dispersion. The C/C samples were then suspended with a wire and immersed in the above three solutions, and the pH was adjusted to 8.5 with dilute ammonia. The immersion solution was refreshed once a day. After 3 days, the samples were removed, cleaned with deionized water, and dried in a Manful oven at 393 K. The treated samples were designated as PDA/C_f_/C, PDA-PVA/C_f_/C, and PDA-PVA-GO/C_f_/C, respectively.

### Coating preparation

A CaP precursor coating was deposited on C_f_/C with PDA, PDA-PVA, and PDA-PVA-GO layers using a homemade HET device, as illustrated in our previous study [33]. The device used a graphite sheet and the pretreated C/C as the anode and cathode. The electrolyte was prepared by mixing 0.16 M Ca(OH)_2_ (Guangdong Guanghua Sci-Tech CO., Ltd., China, AR), 0.18 M H_3_PO_4_ (Wengjiang Chemical Reagent Co., Ltd., China, AR), and 0.45 M lactic acid (Guangzhou Jinyuan Chemical Co., Ltd., China, AR) the pH of which was buffered to 2.5 by a dilute solution of glacial acetic acid (Macklin, Shanghai, AR). The deposition current density was 5 mA/cm^2^, and the deposition duration was 80 min at 363 K. The obtained calcium phosphate sample with a PDA, PDA-PVA, and PDA-PVA-GO layer was designated as CaP/PDA/C_f_/C, CaP/PDA-PVA/C_f_/C, and CaP/PDA-PVA-GO/C_f_/C, respectively. For comparison, the CaP coating without a transition layer was designated as CaP/C_f_/C.

All these CaP coatings were then post-treated in an autoclave with a 35 mL solution of 20% aqueous ammonia (Shanghai Macklin) at 413 K for 24 h. Finally, these post-treated samples were ultrasonically washed with distilled water and dried at 393 K in an oven. The obtained samples transformed from CaP/C_f_/C, CaP/PDA/C_f_/C, CaP/PDA-PVA/C_f_/C, and CaP/PDA-PVA-GO/C_f_/C were recorded as HA/C_f_/C, HA/PDA-PVA/C_f_/C, HA/PDA-PVA/C_f_/C, and HA/PDA-PVA-GO/C_f_/C, respectively.

In addition, samples with the HA coatings were immersed in simulated body fluid (SBF) to evaluate their in vitro bioactivity [34]. The SBF was prepared by dissolving reagents of NaCl, NaHCO_3_, KCl, K_2_HPO_4_·3H_2_O, MgCl_2_·6H_2_O, CaCl_2_, Na_2_SO_4_ into deionized water at a low temperature, and its pH was adjusted to 7.40 with hydrochloric acid. The HA-coated samples were soaked in 30 mL SBF solution at 310 K in centrifugal plastic tubes. The SBF solution was refreshed every day. After soaking for 1, 3, 5, and 7 days, respectively, the samples were removed, rinsed with distilled water, and dried in a Muffle oven for subsequent characterization.

### Characterization methods

The hydrophilicity of the samples was measured using an optical contact angle meter and interfacial tension meter (SL 150B, Shanghai Solon). The morphology and quantitative compositional analyses were characterized by scanning electron microscopy (SEM) (Hitachi, Japan) equipped with energy dispersive spectroscopy (EDS). The phase of the coatings was identified using a D8 Advance X-ray diffractometer (CuKα radiation). A Fourier Transform Infrared (FTIR) spectrometer (Nicolet 6700) was used to identify the functional groups of the samples. The adhesion strength of the HA coatings deposited on C/C was measured using a scratch tester (s-3400 N, CMS Reventest), which was equipped with a Rochwell C 0.2 mm diamond stylus with a preload of 3 N. The scratch trace was observed using an optical microscope and a desktop electron microscope (Hitachi, Japan).

### Cell culture

Mouse embryonic osteoblast precursor (MC3T3-E1) cells were purchased from Shanghai Zhong Qiao Xin Zhou Biotechnology Co., Ltd. MC3T3-E1 cells were cultured in α-MEM (Gibco, USA) containing 10% fetal bovine serum (FBS, ExCell Bio, China), streptomycin (0.1 mg/mL), and penicillin (100 U/mL, Solarbio, China), and incubated in 37 °C incubator with 5% CO_2_. Rat bone marrow mesenchymal stem cells (BMSCs) were purchased from Shanghai FuHeng Biology Co., Ltd. BMSCs were cultured in DMEM (Gibco, USA) under the same conditions as MC3T3-E1. The osteogenic induction medium for BMSCs was supplemented with 2 mM β-glycerophosphate disodium, 100 μM L-ascorbic acid, and 10 nM dexamethasone (Aladdin, China).

### CCK-8 assay

First, HA/C_f_/C, HA/PDA/C_f_/C, HA/PDA-PVA/C_f_/C, and HA/PDA-PVA-GO/C_f_/C were immersed in α-MEM for 48 h to prepare extracts (0.1 g/mL). The MC3T3-E1 cell suspension was inoculated into 96-well plates at a density of 4 × 10^3^ cells/well for 12 h. The medium was then changed to extracts from different samples, and the cells were cultured for 48 h. After culture, 10 μL of CCK-8 solution (Beyotime, China) was added to each well and incubated for 1 h. The whole plate was measured at 450 nm using a spectrophotometer (Epoch, BioTek).

### Cell adhesion and morphological imaging

HA/C_f_/C, HA/PDA/C_f_/C, HA/PDA-PVA/C_f_/C, and HA/PDA-PVA-GO/C_f_/C were sterilized with ethylene oxide. MC3T3-E1 cells at a density of 1 × 10^5^ cells/mL were cultured on the surface of sterilized HA/C_f_/C, HA/PDA/C_f_/C, HA/PDA-PVA/C_f_/C, and HA/PDA-PVA-GO/C_f_/C in the 24-well plates, respectively. Then, the 24-well plates were incubated for 48 h. After 48 h, the cells were stained with DAPI (Beyotime, China). The stained samples were observed under a fluorescence microscope (Axioskop 40, Zeiss). In addition, after the above observation, the samples were also observed under SEM (Thermofisher, QUANTA 250 FEG) and photographed.

In addition, to further observe the growth of MC3T3-E1 cells on the surface of materials, the materials cultured with MC3T3-E1 cells were observed under SEM. The cell morphology on the surface of the materials was observed under SEM and photographed.

### ALP staining

First, BMSCs were plated in 6-well plates (1 × 10^4^ cells/well), marking the start as Day 1. On Day 3, the medium was changed and the groups were arranged as follows: (1) DMEM medium (negative control); (2) the osteogenic induction medium (positive control, PC); (3) the extracts of osteogenic induction medium and HA/C_f_/C; (4) the extracts of osteogenic induction medium and HA/PDA/C_f_/C; (5) the extracts of osteogenic induction medium and HA/PDA-PVA/C_f_/C; (5) the extracts of osteogenic induction medium and HA/PDA-PVA-GO/C_f_/C. The medium was changed twice a day for 1 week. On Day 10, the cells were fixed with 4% paraformaldehyde, stained with the NBT/BCIP substrate solution (Roche Diagnostics GmbH, Germany), and subsequently examined under an inverted fluorescence microscope (iX71, Olympus).

### Statistical analysis

All data are presented as the mean value ± standard deviation of at least three independent measurements. Comparisons between multiple groups were analyzed using the one-way ANOVA test. Statistical analyses were conducted using GraphPad Prism 8. Mean differences with *P* < 0.05 were considered statistically significant. In the figures, asterisks denote statistical significance as follows: *P < 0.05, **P < 0.01, and ***P < 0.001, respectively.

## Results and discussion

### Fabrication of different C_f_/C samples

**Fig. 1:**
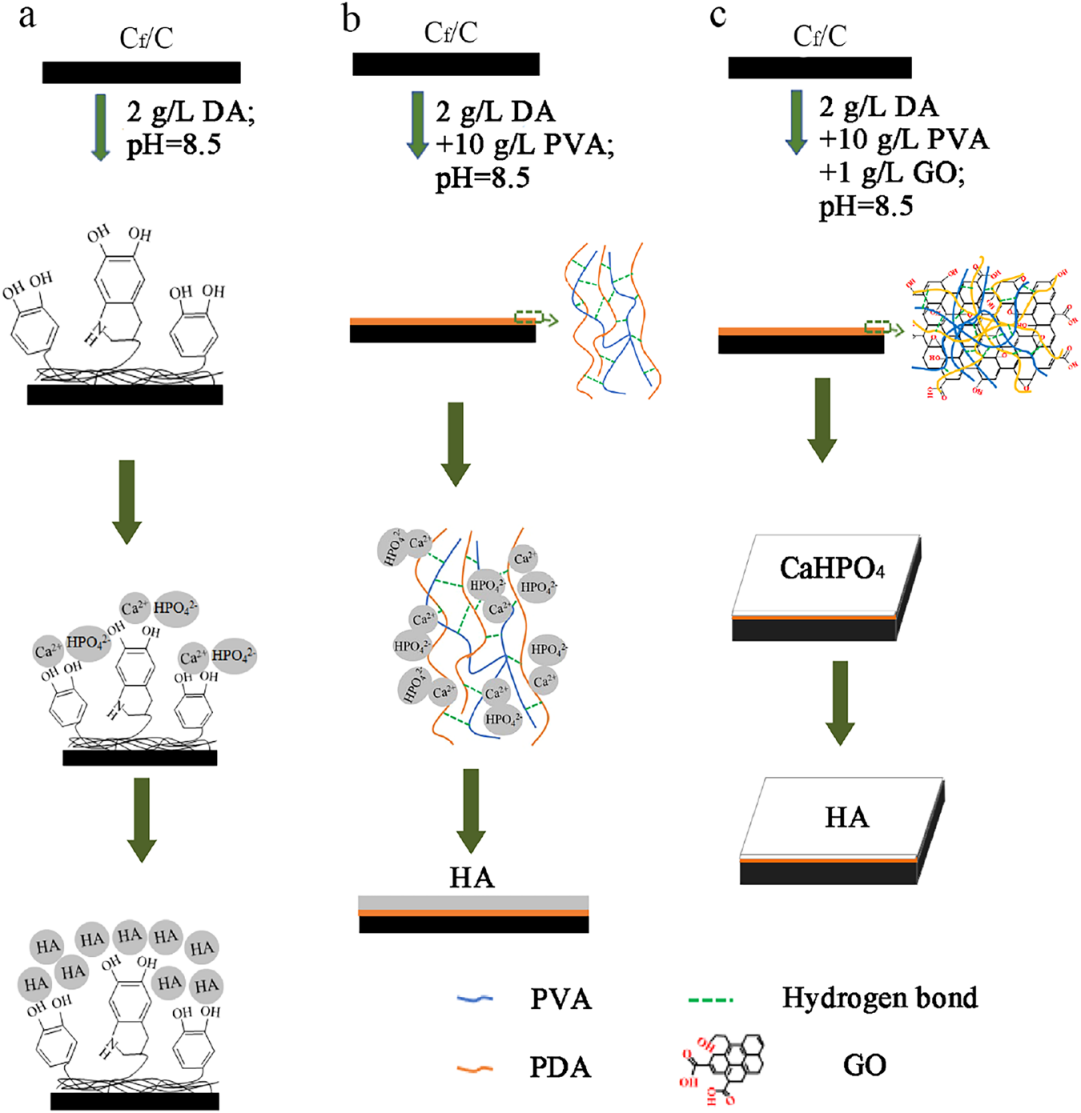
Schematic of specimen preparation procedure: **a** HA/DA/C_f_/C, **b** HA/PDA-PVA/C_f_/C, and **c** HA/PDA-/PVA-GO/C_f_/C

The preparation procedures of HA/PDA/C_f_/C, HA/PDA-PVA/C_f_/C, and HA/PDA-PVA-GO/C_f_/C samples are illustrated in Fig. 1. Initially, the C_f_/C substrate is immersed in DA solution (pH = 8.5), leading to the formation of a PDA film through oxidation and self-polymerization of DA (Fig. 1a). Subsequent immersion of the PDA-coated C_f_/C specimen in an electrolyte containing Ca and P ions within autoclave results in chelation between the phenolic group on PDA and Ca^2+^ ions in the electrolyte. This interaction and the presence of OH- ions from H^+^ reduction facilitate the production of a CaP precursor coating during HED [35], which eventually transforms into an HA coating via post-hydrothermal treatment [36]. The preparation procedure for the DA-PVA transition layer mirrors that of the PDA layer (Fig. 1b), wherein immersion of the C_f_/C substrate in a DA-PVA solution (pH = 8.5) leads to the formation of a PDA-PVA film due to hydrogen bonding between PDA and PVA molecules. During HED and subsequent conversion, Ca^2+^ ions not only chelate with PDA but also engage in ion-dipole interactions with the oxygen atoms in the free -OH groups of the PVA molecule [33], facilitating HA’s nucleation and growth within the PDA-PVA matrix [34]. Figure 1c depicts the procedure for preparing a PDA-PVA-GO layer on the C_f_/C surface, wherein PDA, generated via the self-polymerization of DA, forms hydrogen bonds with both PVA and GO, while also connecting GO through residual catechol groups and oxidized quinone forms of catechol, resulting in a PDA-PVA-GO transition layer. Subsequently, analogous to the formation of HA/PDA/C_f_/C, the final C_f_/C material compositing with the HA/PDA-GO transition layer is achieved through HED and post-hydrothermal treatment.

### Characterization of PDA-PVA-GO/C_f_/C

**Fig. 2:**
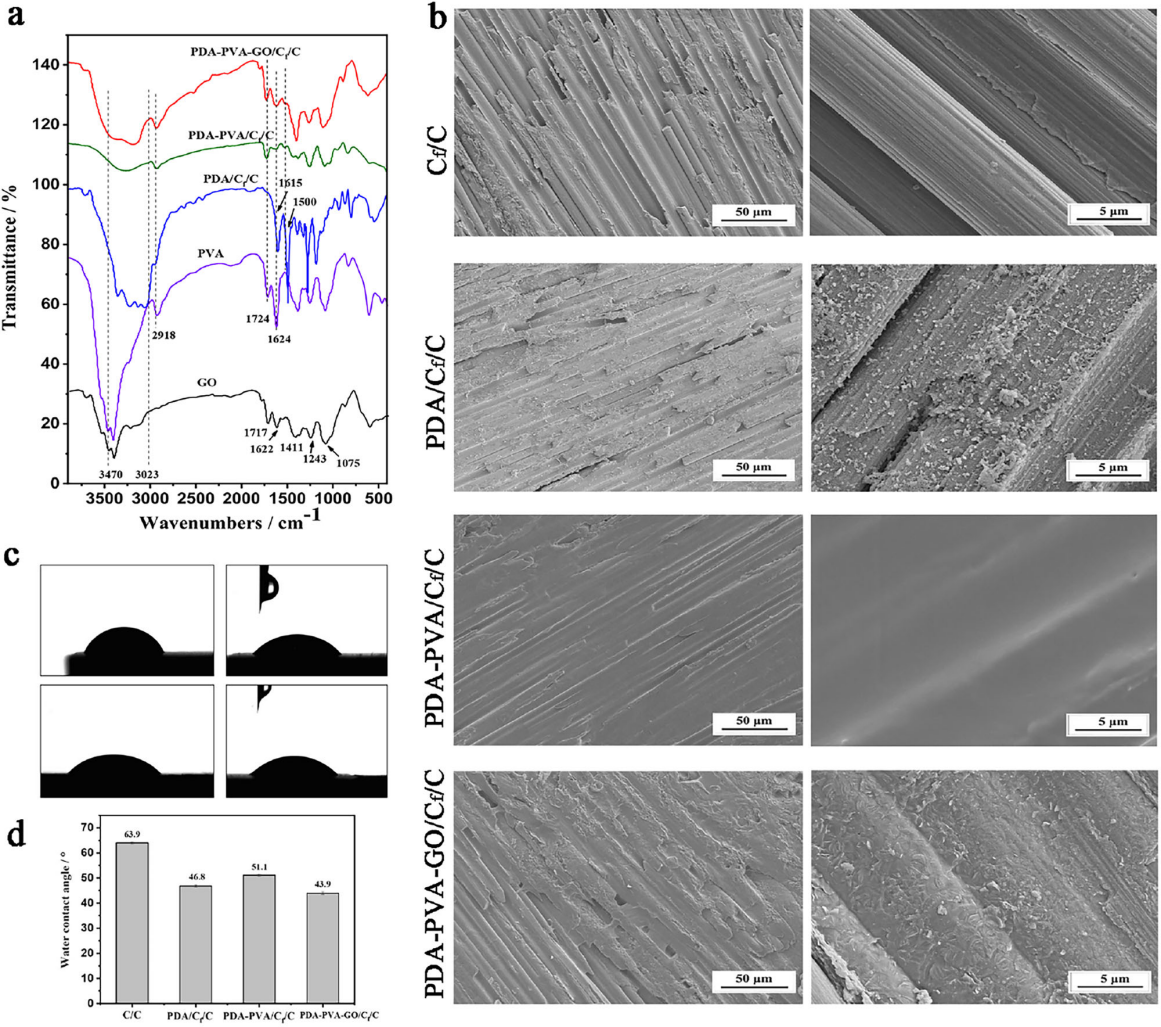
Characterization of PDA/C_f_/C, PDA/PVA/C_f_/C, and PDA-PVA-GO/C_f_/C. **a** FTIR spectra of the transition layer of various samples. **b** SEM images of samples. Scale bars: 50 μm and 5 μm. **c** Water contact angle photographs of C_f_/C (upper left), PDA/C_f_/C (upper right), PDA/PVA/C_f_/C (bottom left), PDA-PVA-GO/C_f_/C (bottom right). **d** Quantitative data of the water contact angle of corresponding samples

The properties of PDA-C_f_/C, PDA-PVA/C_f_/C, and PDA-PVA-GO/C_f_/C samples were tested. First, the surface species collected from the PDA-C_f_/C, PDA-PVA/C_f_/C, and PDA-PVA-GO/C_f_/C samples were characterized using the FTIR spectra (Fig. 2a). To provide context, FTIR spectra of GO and PVA were also analyzed. In the spectrum of GO, the peak at 1717 cm^−1^ corresponds to the C=O stretching vibration peak of the carboxyl functional group at the GO edges, while the peak at 1622 cm^−1^ arises from the C=C stretching vibration between unoxidized graphite layers. Additionally, the spectral peak observed at 1411 cm^−1^ is assigned to the deformation of O–H bonds, while the peak at 1243 cm^−1^ corresponds to the C–O stretching vibration. The C–O–C stretching vibration is manifested at 1075 cm^−1^. Furthermore, the range between 3023–3470 cm^−1^ indicates O–H stretching vibration [37]. Comparatively, in PDA-PVA/C_f_/C and PDA-PVA-GO/C_f_/C spectra, the O–H peaks exhibit a shift to lower wave numbers, likely due to hydrogen bonding interactions among PDA, PVA, and GO [38]. A consistent C–H stretching vibration peak at ~2918 cm^−1^ is observed across all FTIR spectra of PVA, PDA/C_f_/C, PDA-PVA/C_f_/C, and PDA-PVA-GO/C_f_/C [39]. Furthermore, the presence of PVA is confirmed by the peak at 1724 cm^−1^, corresponding to the O–H flexural vibration peak, which aligns with PVA’s signature. Analysis of the FTIR spectrum of PDA/C_f_/C reveals characteristic absorption peaks of the benzene ring at 1615 cm^−1^ and 1500 cm^−1^ [40, 41], indicating the existence of dopamine and polydopamine. Notably, in PDA/C_f_/C and PDA-PVA-GO/C_f_/C spectra, the peaks at 1615 cm^−1^ and 1500 cm^−1^ are shifted to the higher wave numbers. Moreover, overlapping peaks of PDA, PVA, and GO between 1075 cm^−1^ and 1615 cm^−1^ suggest the mutual influence of hydrogen bonding among these components.

The SEM images depicting the morphologies of pristine C_f_/C, PDA/C_f_/C, PDA-PVA/C_f_/C, and PDA-PVA-GO/C_f_/C are displayed in Fig. 2b. The surface of pristine C_f_/C exhibits a rugged texture with visible carbon fibers, alongside small pores and grooves. In contrast, the PDA/C_f_/C surface appears coated with a layered film, with granular PDA evident upon closer inspection. Introducing PVA into the PDA layer results in a smoother surface than PDA/C_f_/C. However, the incorporation of GO into the PDA-PVA layer leads to a return to a rougher surface texture in PDA-PVA-GO/C_f_/C samples. These observations suggest that while adding polymers alters the surface characteristics, the resulting composites can mitigate the influence of the original C_f_/C composite’s diverse carbon structures on the adhesive properties of HA coatings.

The hydrophilic and hydrophobic properties of the material surface play an important role in subsequent calcium phosphate coating deposition. To detect the surface hydrophilic and hydrophobic properties of materials, water contact angle tests were conducted on pristine C_f_/C, PDA/C_f_/C, PDA-PVA/C_f_/C, and PDA-PVA-GO/C_f_/C samples (Fig. 2c, d). The water contact angle of bare C_f_/C measures 63.9°, while those of PDA/C_f_/C, PDA-PVA/C_f_/C, and PDA-PVA-GO/C_f_/C are 46.8°, 51.1°, and 43.9°, respectively. The consistent decrease in contact angle across the PDA, PDA-PVA, and PDA-PVA-GO layers suggests an enhancement in the surface hydrophobicity of C_f_/C after applying PDA or PVA, facilitating subsequent calcium phosphate coating deposition. Notably, the water contact angle for PDA-PVA/C_f_/C surpasses that of both PDA/C_f_/C and PDA-PVA-GO/C_f_/C, possibly attributable to its smoother surface morphology.

### Characterization of CaP/PDA-PVA-GO/C_f_/C

**Fig. 3:**
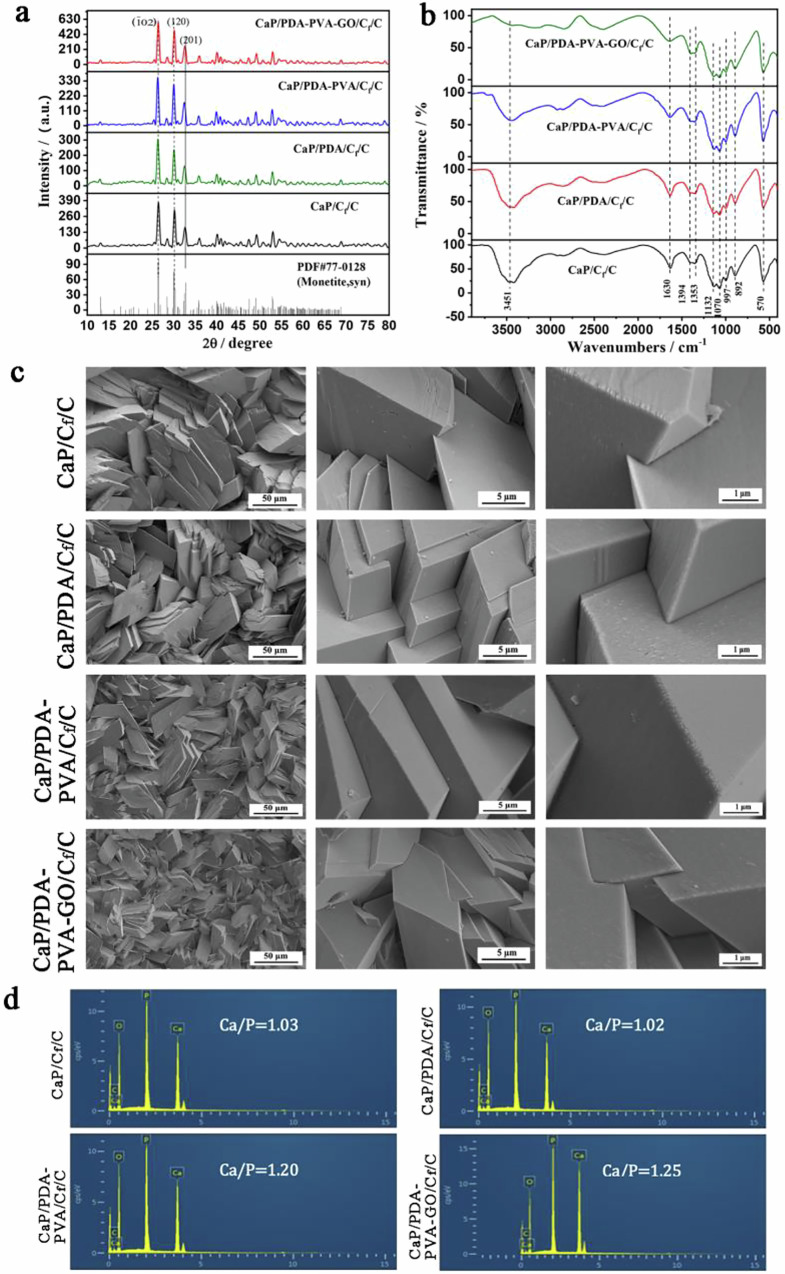
Characterization of CaP/C_f_/C, CaP/PDA/C_f_/C, CaP/PDA/PVA/C_f_/C, and CaP/PDA-PVA-GO/C_f_/C. **a** XRD patterns for different samples. **b** FTIR spectra of different samples. **c** SEM images of samples. Scale bars: 50 μm, 5 μm, and 1 μm. **d** EDS spectra of various samples

The properties of CaP precursor coatings, prepared via HED, were tested on naked C_f_/C, PDA/C_f_/C, PDA/PVA/C_f_/C, and PDA-PVA-GO/C_f_/C substrates. For convenience, we denote the samples with bare C_f_/C, PDA/C_f_/C, PDA-PVA/C_f_/C, and PDA-PVA-GO/C_f_/C as CaP/C_f_/C, CaP/PDA/C_f_/C, CaP/PDA-PVA/C_f_/C, and CaP/PDA-PVA-GO/C_f_/C, respectively. The XRD diffraction patterns of different coatings are illustrated in Fig. 3a. Notably, characteristic diffraction peaks at ~26.50°, 30.09°, and 32.44°, corresponding to crystal planes (102), (120), (201) of CaHPO_4_ (monoclinic monetite phase, JCPDS 77-0128, monetite, syn), respectively, are prominently observed. These peaks, along with all other indexed peaks, signify the prevalence of the monoclinic monetite phase across all coatings. Furthermore, a subtle presence of peaks at (102) and (201) suggests partial incorporation of PDA and PVA into the monetite lattice. However, for the PDA-PVA-GO/C_f_/C composites, the diffraction peak positions of monetite closely resemble those of the pristine C_f_/C composite. This observation indicates that the incorporation of GO into PDA-PVA may hinder its integration into the monetite lattice.

Figure 3b exhibits the FTIR spectra of CaP/C_f_/C, CaP/PDA/C_f_/C, CaP/PDA-PVA/C_f_/C, and CaP/PDA-PVA-GO/C_f_/C. The bands at 3451 cm^−1^ and 1630 cm^−1^ are attributed to the stretching and bending vibrations of adsorbed water [42], respectively. Noteworthy characteristic absorption peaks of PO_4_^3−^ emerge at 1132 cm^−1^, 1070 cm^−1^, 997 cm^−1^ and 570 cm^−1^ [43]. Additionally, the peak at 892 cm^−1^ corresponds to the vibrational peak of HPO_4_^2−^ [44]. Notably, vibrations at 1394 cm^−1^ and 1353 cm^−1^ [45] are observed, originating from the CO_3_^2−^ group. This group is introduced through the dissolution of CO_2_ in air within the electrolyte, subsequently replacing part of the PO_4_^3−^ in the monetite lattice.

The SEM morphologies of the CaHPO_4_ coatings are depicted in Fig. 3c. Notably, there is a minimal disparity in the configuration of the four precursor coatings, all presenting irregular tetragonal crystals interlocked in structure. This morphology suggests an enhanced bonding performance of the coatings. Upon closer inspection at high magnification, the size of the block crystals diminishes in the following order: CaP/C_f_/C, CaP/PDA/C_f_/C, CaP/PDA-PVA/C_f_/C, and CaP/PDA-PVA-GO/C_f_/C samples. The corresponding EDS spectra, as illustrated in Fig. 3d, reveal the presence of Ca and P elements originating from the coatings. In contrast, C elements derive from the C/C matrix or the transition layer of PDA, PDA-PVA, and PDA-PVA-GO. The Ca/P atomic ratios of these samples are 1.03, 1.02, 1.20, and 1.25, respectively. Notably, the CaP/C_f_/C and CaP/PDA/C_f_/C coatings exhibit similar Ca/P atomic ratios close to the theoretical value of the monetite phase, whereas the CaP/PDA-PVA/C_f_/C and CaP/PDA-PVA-GO/C_f_/C coatings demonstrate a Ca/P atomic ratio exceeding the theoretical value of monetite, potentially due to the higher solubility of PVA compared with PDA, which may induce the deposition of more Ca ions.

### Characterization of HA/PDA-PVA-GO/C_f_/C

**Fig. 4:**
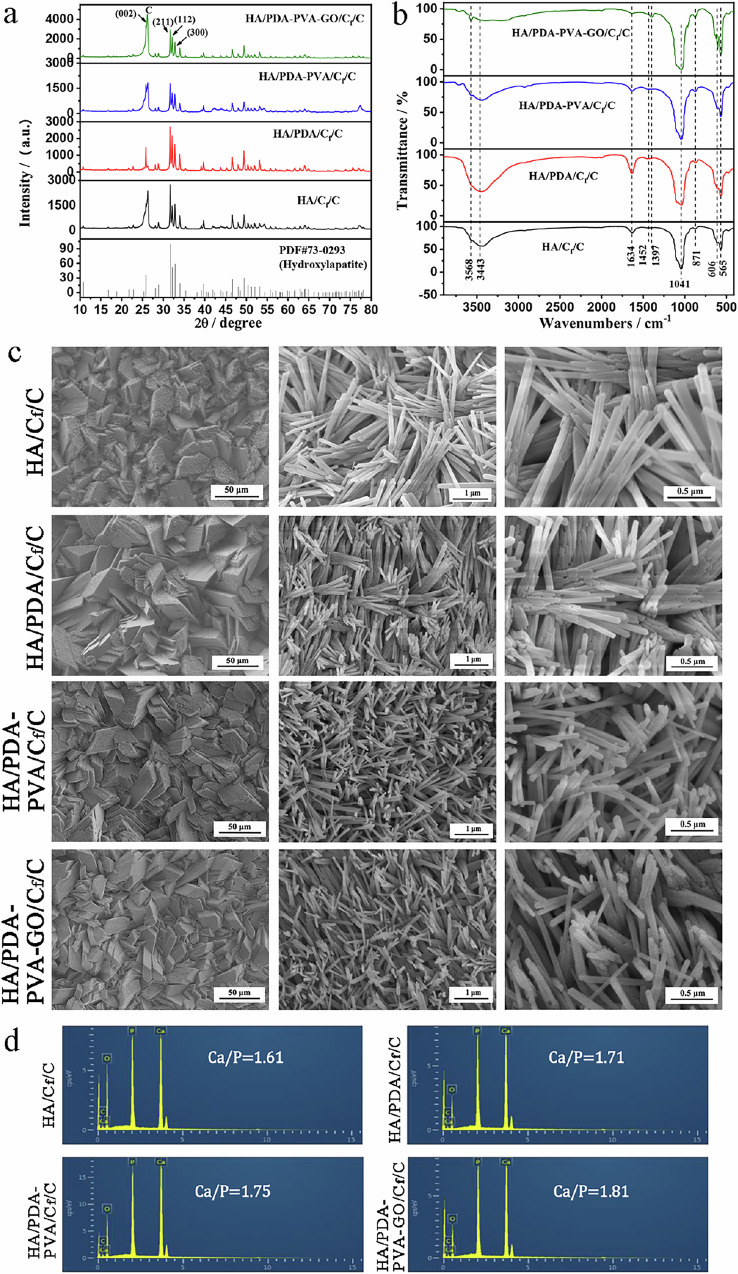
Characterization of HA/C_f_/C, HA/PDA/C_f_/C, HA/PDA-PVA/C_f_/C, and HA/PDA-PVA-GO/C_f_/C. **a** XRD patterns for different samples. **b** FTIR spectra of different samples. **c** SEM images of various samples. Scale bars: 50 μm, 1 μm, and 0.5 μm. **d** EDS spectra of various samples

The properties of the coatings transformed from the CaHPO_4_ coatings on C_f_/C, PDA-C_f_/C, PDA-PVA/C_f_/C, and PDA-PVA-GO/C_f_/C substrates via post-hydrothermal treatment were tested. Figure 4a presents the XRD patterns of various coatings. Except for the carbon diffraction peak at around 26°, all the other peaks correspond to HA (JCPDS 73-0293), indicating successful conversion from CaHPO_4_ to HA coatings with good crystallinity. Interestingly, unlike the monetite coating, no peak shift is observed for the HA coating, suggesting that PVA in the monetite coating does not affect the HA lattice post-hydrothermal treatment.

Figure 4b displays the FTIR spectra of the obtained HA coatings transformed from CaHPO_4_ coatings deposited on various substrates. The distinctive diffraction peak of HA, arising at 3568 cm^−1^, is attributed to the stretching vibrations of the O–H bonds [46]. Additionally, peaks attributed to adsorbed water [42], PO_4_^3−^ [43], and CO_3_^2−^ [47] are observed. Notably, HA coatings with PVA species, especially with simultaneous PVA and PDA, exhibit stronger OH- peaks and more CO_3_^2−^ in the lattice. Moreover, no additional groups corresponding to PDA are found, indicating that PDA primarily exists between the HA external coating and the C/C matrix.

The SEM images in Fig. 4c depict HA coatings retaining an interlinked tetragonal bulk similar to their CaHPO_4_ precursor, implying an in-situ transformation mechanism from monetite to HA [48]. However, needle-like HA crystal clusters are observed within the HA bulk, forming an interlaced structure. The size decrease of HA crystals is most significant for coatings without a transition layer, followed by those with PDA, PDA-PVA, and PDA-PVA-GO layers. This may be because PDA and PVA offer increased nucleation sites, facilitating the crystallization of HA crystals. In addition, the needle-like crystals of the HA/PDA-PVA-GO coating stack to be the densest with a greater degree of entanglement, leaving the fewest pores. The EDS spectra (Fig. 4d) reveal higher Ca/P atomic ratios for HA, HA/PDA, HA/PDA-PVA, and HA/PDA-PVA-GO coatings, possibly due to their higher CO_3_^2–^ content compared to HA coatings.

### Cross-section analysis and mechanical performance of HA/C_f_/C, HA/PDA/C_f_/C, HA/PDA-PVA/C_f_/C, and HA/PDA-PVA-GO/C_f_/C

**Fig. 5:**
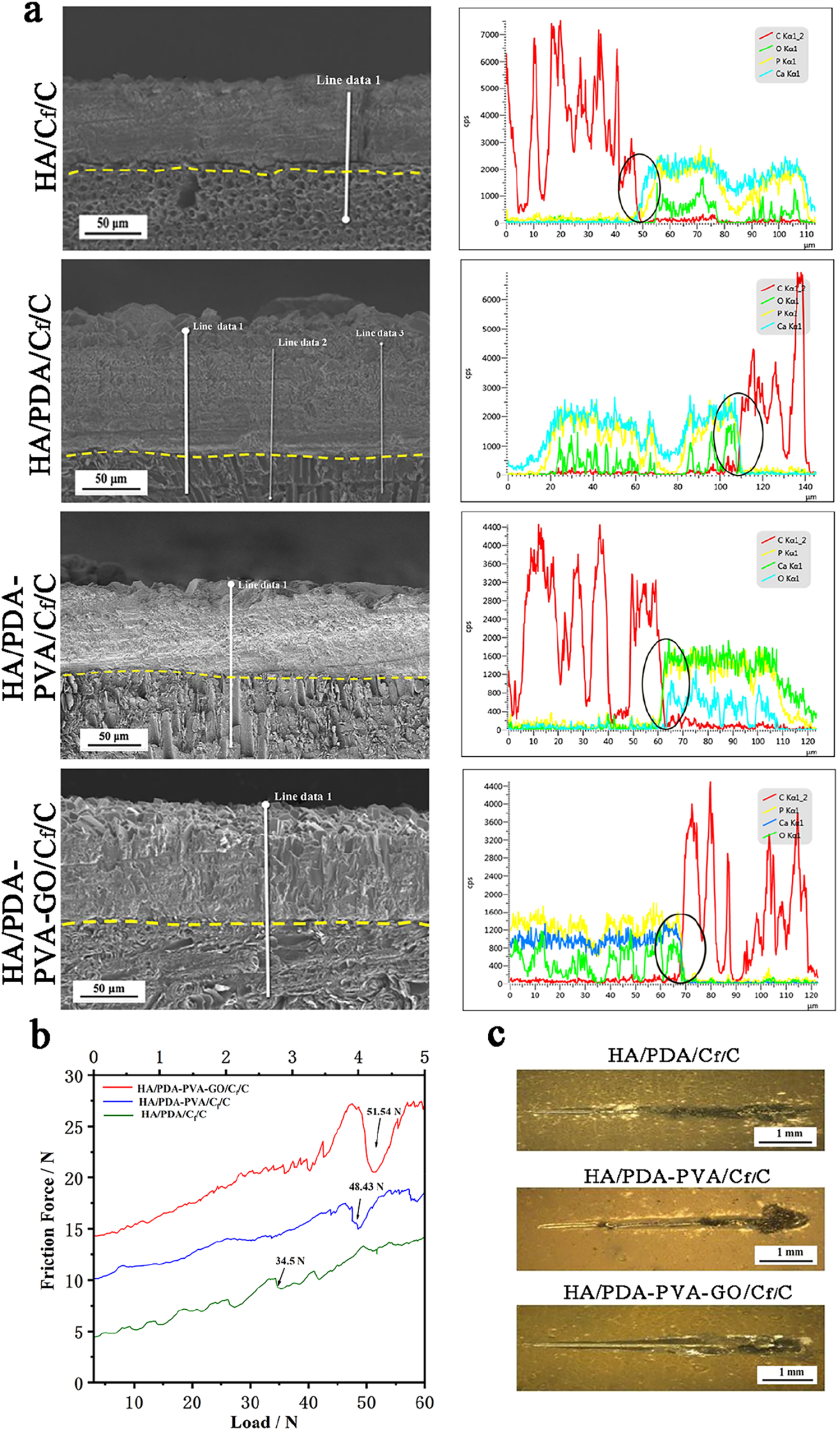
Cross-section analysis and mechanical performance of HA/C_f_/C, HA/PDA/C_f_/C, HA/PDA-PVA/C_f_/C, and HA/PDA-PVA-GO/C_f_/C. **a** Cross-section profiles and EDS line scan of different samples. Scale bar = 50 μm. **b** Scratch curves of different samples. **c** Scratch trace of different samples. Scale bar = 1 mm

The Cross-section analysis and mechanical performance test of HA/C_f_/C, HA/PDA/C_f_/C, HA/PDA/PVA/C_f_/C, and HA/PDA-PVA-GO/C_f_/C were conducted. Figure 5a displays the cross-sectional profiles and elemental linear distribution diagrams of HA/C_f_/C, HA/PDA/C_f_/C, HA/PDA-PVA/C_f_/C, and HA/PDA-PVA-GO/C_f_/C samples. Visible interfacial gaps between the HA coating and the Cf/C substrate (indicated by the yellow dashed line in Fig. 5a) are attributed to the insufficient bonding at the HA-Cf/C interface, which induces preferential crack initiation during the polishing process. Subsequently, these cracks propagate along the interface, leading to the formation of visible gaps. However, the HA/PDA, HA/PDA-PVA, and HA/PDA-PVA-GO specimens exhibit dense cross-sectional morphologies devoid of obvious gaps and pores, suggesting that the transition layers can fill defects between them, achieving a dense interface with the HA coating and the C_f_/C composites. Additionally, these layers, being elastic polymers, act as buffers, preventing crack propagation in the interface.

The elemental linear distribution diagram corroborates these findings. A distinct interface of elemental composition emerges between the C element from the C_f_/C matrix and the Ca, P, and O elements from the coatings (as depicted by the black circle in Fig. 5a), consistent with the cross-section morphology. Conversely, for the HA/PDA/C_f_/C, HA/PDA-PVA/C_f_/C, and HA/PDA-PVA-GO/C_f_/C samples, the C element overlaps with the elements of Ca, P, and O elements at the interface of the C/C substrate and the coatings, indicating the penetration of Ca and P ions into the PDA, PDA-PVA, and PDA-PVA-GO interlayers, forming a composite interface of HA, PDA, PVA, and GO. These phenomena suggest that the HA coating with the polymer interface exhibits superior mechanical performance.

The mechanical performance of HA/PDA, HA/PAD-PVA, and HA/PDA-PAV-GO coatings was evaluated through a scratch test, with corresponding scratch curves and traces shown in Fig. 5b, c. The critical load of HA/PDA, HA/PAD-PVA, and HA/PDA-PAV-GO coatings, determined by the peel point of the coatings, is 34.5, 48.43, and 51.54 N, respectively. This indicates that the addition of the polymer transition layer of PDA and PVA, especially GO, strengthens the HA interface and thus improves the adhesion. Accordingly, the shear strength of the HA/PDA, HA/PDA-PVA, and HA/PDA-PVA-GO coatings was calculated to be 99.26, 117.61, and 121.32 MPa, respectively, according to the following expression [42]:

$${\tau }_{C}={({H}_{S}{L}_{C}/\pi )}^{1/2}/R$$

Where *τ*_*c*_ is the shear stress, *R* is the radius of the diamond stylus, *L*_*c*_ is the critical load, and *H*_*s*_ is the Shore scleroscope hardness of the C_f_/C substrate. From the optical microscope, the overall scratch of these coatings before failure is smooth and flat, and no apparent avalanche phenomenon occurs, indicating their superior bonding performance. The exceptional critical load (*L*_*c*_ = 51.54 N) and calculated shear strength (*τ*_*c*_ = 121.32 MPa) for HA/PDA-PVA-GO/C_f_/C significantly surpass reported tensile bond strengths for HA coatings on C_f_/C using SiC (12.56 MPa [17]) or CNT (11.14 MPa [8]) transition layers. While the numerical difference is partly attributed to the distinct test methods (scratch-derived shear vs. tensile pull-off), the high *L*_*c*_ value itself demonstrates superior interfacial adhesion, ranking among the best for electrochemically prepared HA coatings. This performance stems fundamentally from the unique PDA-PVA-GO interphase. PDA ensures robust substrate adhesion. PVA provides toughness and facilitates strong interactions with HA. Crucially, EDS analysis (Fig. 5a) reveals elemental interpenetration (Ca, P, O into the transition zone), indicating the formation of a true composite interface where HA intimately binds within the polymer matrix, far exceeding the primarily mechanical anchoring of SiC/CNT layers. GO reinforces the matrix, hinders cracks, stabilizes PVA, and enhances cohesion. This synergistic combination creates a toughened, integrated interphase, effectively mitigating interfacial stresses.

### Characterization of HA/C_f_/C, HA/PDA/C_f_/C, HA/PDA/PVA/C_f_/C, and HA/PDA-PVA-GO/C_f_/C

**Fig. 6:**
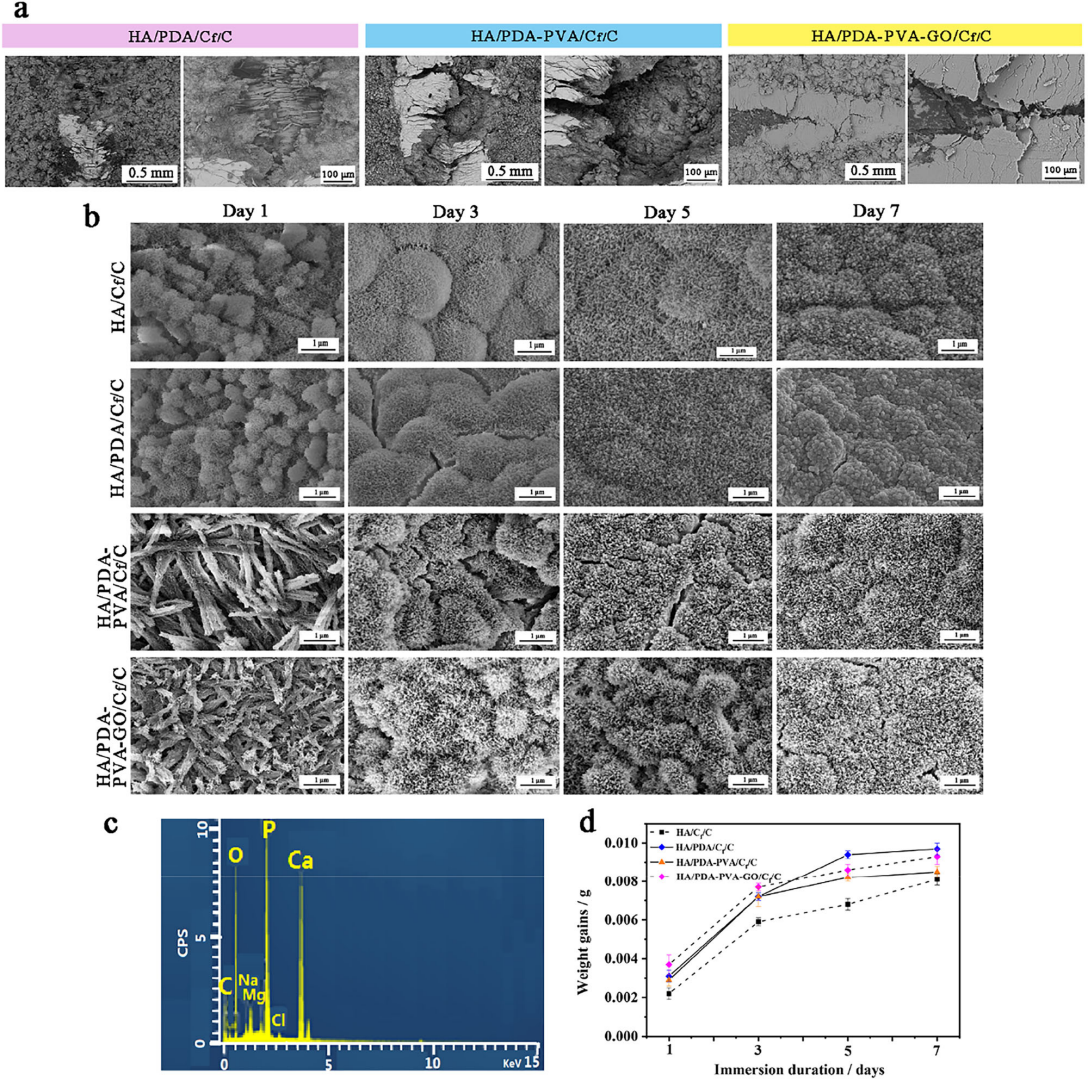
Characterization of HA/C_f_/C, HA/PDA/C_f_/C, HA/PDA-PVA/C_f_/C, and HA/PDA-PVA-GO/C_f_/C. **a** SEM images of coating failure sites in various samples. Scale bars: 0.5 mm and 100 μm. **b** SEM images of various specimens after immersion in SBF solution for 1, 3, 5, and 7 days were obtained. Scale bar: 1 μm. **c** Energy spectrum of spheroid-like deposits. **d** Weight gains of various samples following immersion in SBF solution for durations of 1, 3, 5, and 7 days were obtained

Further SEM observation of the failure site of the coatings (Fig. 6a) reveals that the HA coating with the PDA layer tends to be stripped from the C_f_/C substrate, exposing naked carbon fibers. However, the exposed area for the HA-PDA coating is lower, indicating an improvement in interface performance, albeit limited. In contrast, the HA coating with the added PVA layer exhibits a distinct behavior. Unlike the delamination observed in the HA-PDA coatings, no carbon fiber or HA coating fragments are exposed, as evidenced in Fig. 6a, underscoring its strong cohesion. Even when subjected to critical loads, the coating residues firmly adhere to the C_f_/C substrate, demonstrating the superior toughness of PVA compared with PDA. However, despite this resilience, sudden detachments are still observed, particularly when the failure site is near the interface zone. This detachment can be ascribed to the compositional dominance of PVA over PDA in the composite. Remarkably, adding GO to the PDA-PVA composite yields even more promising results. Not only does the scratch trace appear smoother than other composites, but also, no sudden detachment is observed. This favorable outcome can be attributed to the remarkable toughening effect of GO over PDA and PVA polymers. Additionally, the presence of GO obstructs the dissolution of PVA into the solution during the post-hydrothermal treatment, further enhancing the cohesion of the coating.

The bioactivity of these HA coatings was assessed following immersion in SBF for varying durations, with corresponding surface morphologies depicted in Fig. 6b. After 1 day of immersion, both the HA and HA/PDA coatings exhibit complete coverage with spheroid-like attachments, indicative of typical bone-like apatite morphology, thus confirming their strong bioactivity. However, the surfaces of the HA/PDA-PVA and HA/PDA-PVA-GO coatings maintain their initial configuration of needle crystals, with numerous attachments visible on their surfaces. This suggests that the needle-shaped crystals have undergone dissolution and recrystallization processes. Subsequently, after 3–7 days of immersion, fluffy spherical protrusions cover the surface morphologies of all four coatings, with an expanding presence over time. Energy spectrum analysis of these spheroid deposits (Fig. 6c) discloses the incorporation of elements including Ca, O, P, Na, Cl, and Mg, further confirming their composition as bone-like apatite.

The weight gain of the coatings over 1, 3, 5, and 7 days of immersion in SBF solution is presented in Fig. 6d. Overall, the weight gain of all coatings exhibits an upward trend with increasing immersion duration. The HA/PDA, HA/PDA-PVA, and HA/PDA-PVA-GO coatings show greater weight gains than the HA coatings, indicating enhanced bioactivity. Among these, the HA/PDA coating exhibits the highest total weight gain, signifying its superior bioactivity, followed by the HA/PDA-PVA-GO coating. In contrast, the HA/PDA-PVA coating displays the least weight gain. This trend may be attributed to the varying amounts of polymer incorporated into the HA coatings.

### In vitro proliferation promotion and osteogenic differentiation

**Fig. 7:**
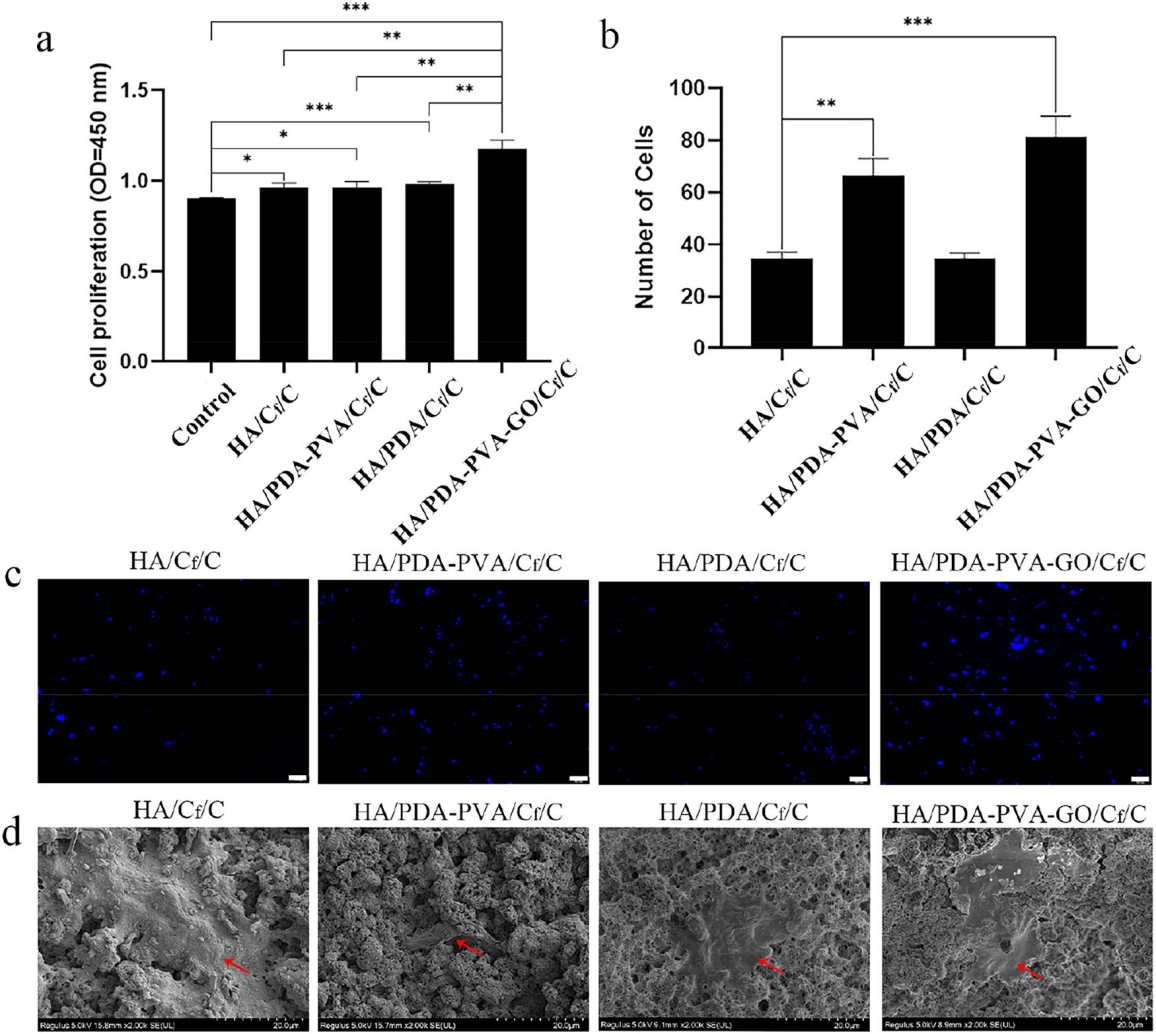
Proliferation of MC3T3-E1 in different groups. **a** CCK-8 assay of extracts from different coatings on the proliferation of MC3T3-E1 cells. **b** Number of cells adhering to the coatings. **c** Fluorescent photographs of MC3T3-E1 cells adhering to coatings. Blue: nuclear staining. Scale bar = 100 μm. **d** SEM photographs of MC3T3-E1 cells adhering to coatings. The red arrows indicate the cells. Scale bar = 20 μm

To evaluate the influence of various coatings on osteoblast proliferation, the viability of cells treated with various coatings was assessed. Initially, MC3T3-E1 cells were utilized as model cells and exposed to extracts of HA/C_f_/C, HA/PDA/C_f_/C, HA/PDA-PVA/C_f_/C, and HA/PDA-PVA-GO/C_f_/C. Following a 48-h co-culture period, cell viability was assessed using the CCK-8 assay. As depicted in Fig. 7a, each group exhibited distinct effects on promoting MC3T3-E1 cell proliferation. Notably, the HA/PDA-PVA-GO/C_f_/C scaffold demonstrated the most significant ability to enhance cell proliferation, surpassing the HA/C_f_/C, HA/PDA/C_f_/C, and HA/PDA-PVA/C_f_/C groups.

Furthermore, cells were cultured with HA/C_f_/C, HA/PDA/C_f_/C, HA/PDA-PVA/C_f_/C, and HA/PDA-PVA-GO/C_f_/C for 48 h without utilizing extracts, and the nuclei of MC3T3-E1 cells on the coating surface were stained. The results (Fig. 7b, c) revealed a higher number of surface cells in the HA/PDA-PVA/C_f_/C and HA/PDA-PVA-GO/C_f_/C groups. Specifically, the HA/PDA-PVA-GO/C_f_/C group exhibited the highest number of surface cells at 81, followed by the HA/PDA-PVA/C_f_/C group at 66 cells. This indicates that MC3T3-E1 cells exhibited superior adhesion and proliferation on the surface of HA/PDA-PVA-GO/C_f_/C. SEM results (Fig. 7d) illustrated visible cell adhesion on the flat surface of HA/PDA-PVA/C_f_/C. However, the surface of the HA/PDA-PVA/C_f_/C coating appeared too rough, with cells observed seeping into the coating pores. This phenomenon could be attributed to the beginning detachment of the HA/PDA-PVA/C_f_/C coating, further confirming its weaker layer adhesion than HA/PDA-PVA-GO/C_f_/C.

**Fig. 8:**
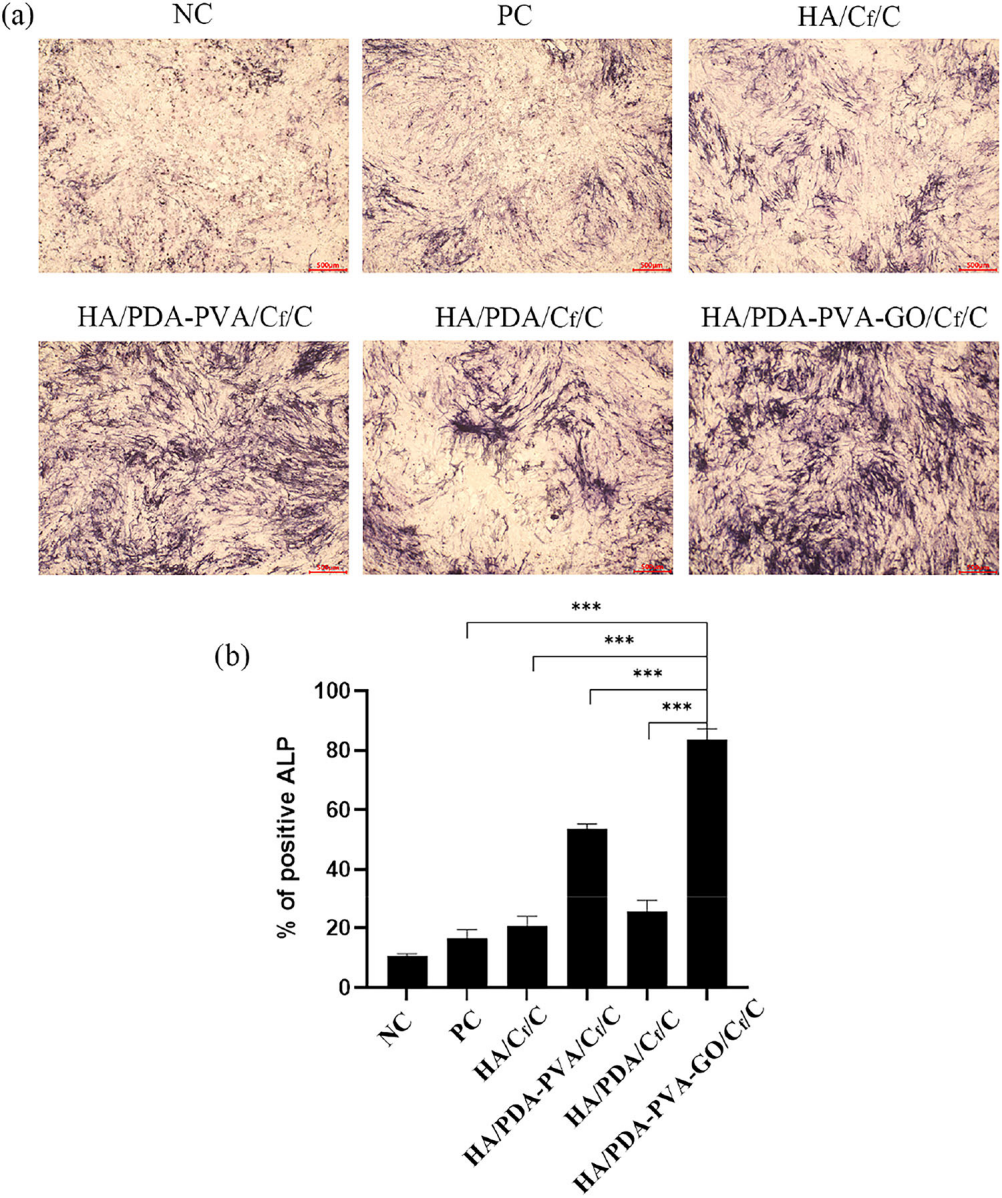
ALP staining of BMSCs in different groups. **a** ALP staining of BMSCs in various groups on day 10. **b** Semi-quantitative results of ALP staining in various groups. Scale bar = 500 μm. ***P < 0.001

To investigate the osteogenic impact of different coatings on osteoblasts, we employed BMSCs as the model cell and examined the ALP formation of BMSCs following a 7-day incubation with extracts of various coatings. As depicted in Fig. 8a, b, HA/C_f_/C, HA/PDA/C_f_/C, HA/PDA-PVA/C_f_/C, and HA/PDA-PVA-GO/C_f_/C, all demonstrated favorable effects on bone regeneration, as evidenced by the elevated ALP expression levels in BMSCs. Notably, the HA/PDA-PVA-GO/C_f_/C group exhibited the most notable performance, showcasing the highest level of positive ALP (83.6%) compared to the HA/C_f_/C (20.7%), HA/PDA-PVA/C_f_/C (53.7%), and HA/PDA/C_f_/C (25.7%) groups.

In general, HA/PDA-PVA-GO/C_f_/C exhibits inherent stability: PDA maintains long-term adhesion via metal-chelation/π-π stacking with GO; crosslinked PVA resists dissolution (supported by FTIR H-bonding); GO’s oxidative stability is enhanced by integration into the network. The dense HA coating serves as a protective barrier, shielding the interlayer. Continuous apatite growth in SBF suggests bioactive sealing may further isolate the transition layer. Scratch tests confirm cohesive failure within the transition layer, proving robust multi-scale bonding resistant to physiological stress. Ongoing work includes accelerated aging studies (PBS, 6–12 months) and planned long-term animal implantation trials to validate in vivo performance. While awaiting long-term data, the component stability, adhesion mechanisms, and HA protection collectively indicate promising durability. HA/PDA-PVA-GO/C_f_/C has significant application prospects in the field of orthopedic implants

## Conclusions

The PDA-PVA-GO/C_f_/C was successfully synthesized using the hydrothermal electrodeposition/post-hydrothermal method. HA/PDA-PVA-GO coatings had higher critical loads than HA/PDA, HA/PDA-PVA, and HA coatings without the transition layer, which is the best among the reported electrochemically prepared HA coatings. Moreover, the HA/PDA-PVA-GO coating exhibited the smoothest scratch trace with no observed sudden delamination from the matrix. HA/PDA, HA/PDA-PVA, and HA/PDA-PVA-GO coatings were more effective in inducing spheroid apatite growth compared to the HA coating alone in vitro. Notably, HA/PDA-PVA-GO/C_f_/C demonstrated superior efficacy in enhancing the proliferation of MC3T3-E1 cells and significantly promoted the ALP production of BMSCs, thereby promoting osteogenesis. These findings suggest that HA/PDA-PVA-GO/C_f_/C represents a promising biomaterial for future clinical bone regeneration.

## Data Availability

All data generated or analyzed in this study are included in this published article.
